# Draft Genome Sequence of the Lichen-Forming Fungus Ramalina intermedia Strain YAF0013

**DOI:** 10.1128/genomeA.00478-18

**Published:** 2018-06-07

**Authors:** Yi Wang, Xiaolong Yuan, Li Chen, Xinyu Wang, Changtian Li

**Affiliations:** a Key Laboratory of Forest Plant Cultivation and Utilization, Yunnan Academy of Forestry, Kunming, China; b Key Laboratory for Plant Diversity and Biogeography of East Asia, Kunming Institute of Botany, Chinese Academy of Sciences, Kunming, China; c Engineering Research Center of the Chinese Ministry of Education for Edible and Medicinal Fungi, Jilin Agricultural University, Changchun, China

## Abstract

Here, we report a draft genome sequence of Ramalina intermedia strain YAF0013. The functional annotation of R. intermedia provides important information related to its ability to produce secondary metabolites. The genome sequence reported here builds the basis for further genome mining.

## GENOME ANNOUNCEMENT

Lichens are stable and self-supporting symbioses between fungi (lichen-forming fungi [LFF]) and photoautotrophic algal partners, namely, green algae and/or cyanobacteria (1). Lichen can produce many unique secondary metabolites that have bioactive, antioxidant, antibacterial, and anticancer properties. Therefore, lichens are an important potential source for pharmacological compounds and the discovery of new drugs (2–5).

R. intermedia belongs to the family Ramalinaceae, and the extracts of Ramalina lichens have shown bioactivity and antioxidant and antibacterial properties (6, 7). R. intermedia also produces several bioactive compounds, such as usnic acid, sekikaic acid, and atranorin. Moreover, a recent report showed that liquid culture of the lichen-forming fungus (LFF) R. intermedia had a broad spectrum of bioactive and antibacterial properties (8).

Currently, there are genomes of five LFF available in the NCBI database, including Caloplaca flavorubescens, Cladonia macilenta, Cladonia metacorallifera, Endocarpon pusillum, and Umbilicaria muehlenbergii (9–13). This is the first genome-sequencing report for a member of the genus Ramalina, which will allow whole-genome sequence comparisons between Ramalina spp. and other LFF with a particular focus on clusters of genes involved in the biosynthesis of bioactive lichen substances.

The LFF strain R. intermedia YAF0013 was isolated from a rock from the Ailao Mountains in southwest China (24.25°N, 101.03°E) in 2013. DNA was extracted using a DNeasy minikit (Qiagen, Valencia, CA, USA). Draft sequencing was performed with the Illumina MiSeq platform using a whole-genome shotgun strategy (Personal Biotechnology Co., Ltd., Shanghai, China). Paired-end reads of 250 bp (13.33 Gb; average coverage, 290×) were assembled using the A5-miseq pipeline (14). The draft nuclear genome of R. intermedia consists of 198 sequence scaffolds with a total assembly length of 26,246,405 bp (*N*_50_, 273,318 bp; *N*_90_, 77,274 bp), a GC content of 51.89%, a maximum scaffold size of 898,913 bp, and 196 contigs greater than 500 bp in size. The completeness of the assembly was assessed using CEGMA (15), which estimated the genome sequence to be 97.58% complete. Subsequent gene prediction analysis using AUGUSTUS version 3.03, GlimmerHMM version 3.0.1, and SNAP version 2006-07-28 (16–18) yielded a total of 8,871 protein-coding genes. The predicted genes were used as queries in searching the nonredundant (NR), eggNOG, KEGG, Swiss-Prot, and Gene Ontology (GO) databases; we predicted 81 cytochrome P450 genes and 30 putative polyketide synthase (PKS) genes, which contained ketoacyl synthase, acyltransferase, and acyl carrier domains. These 30 putative PKSs include 12 nonreducing PKS genes, 17 highly reducing PKS genes, and 1 partially reducing PKS (19).

### Accession number(s).

The draft genome sequence of R. intermedia YAF0013 has been deposited in DDBJ/EMBL/GenBank under the accession no. PEKF00000000 (BioProject SAMN07955959). The version described in this article is the first version, PEKF01000000.
